# Ethical challenges in home mechanical ventilation: A secondary analysis

**DOI:** 10.1177/0969733011414967

**Published:** 2012-03

**Authors:** Knut Dybwik, Erik Waage Nielsen, Berit Støre Brinchmann

**Affiliations:** Nordland Hospital, Norway; Nordland Hospital, Norway; University of Nordland, Norway; University of Tromsø, Norway; University of Nordland, Norway

**Keywords:** autonomy, beneficence, ethical challenges, home mechanical ventilation, justice, non-maleficence

## Abstract

The aim of this study was to explore the ethical challenges in home mechanical ventilation based on a secondary analysis of qualitative empirical data. The data included perceptions of healthcare professionals in hospitals and community health services and family members of children and adults using home mechanical ventilation. The findings show that a number of ethical challenges, or dilemmas, arise at all levels in the course of treatment: deciding who should be offered home mechanical ventilation, respect for patient and family wishes, quality of life, dignity and equal access to home mechanical ventilation. Other challenges were the impacts home mechanical ventilation had on the patient, the family, the healthcare services and the allocation of resources. A better and broader understanding of these issues is crucial in order to improve the quality of care for both patient and family and assist healthcare professionals involved in home mechanical ventilation to make decisions for the good of the patient and his or her family.

## Background

In respiratory medicine, some of the most difficult ethical decisions involve patients with end stage of respiratory failure^1^ and specifically when therapy choices become a question of life or death.^2^ This is especially the case with fast progressing and incurable neuromuscular conditions such as amyotrophic lateral sclerosis (ALS)^3^ and children with spinal muscle atrophy (SMA) type 1.^4^ In these cases, the prognosis is extremely poor and respiratory complications contribute to hasty death if life-sustaining mechanical ventilation is not initiated. The increasing use of home mechanical ventilation (HMV), especially during the last two decades, has shown that advanced treatment and extensive care can keep these patients alive for many years. The use of HMV raises, however, a number of controversial ethical issues. Some of the classic issues include patient autonomy versus physician paternalism, beneficence and non-maleficence, withholding and withdrawing life-prolonging treatment and distributive justice.^5^ The ethical challenges related to this subject are primarily described in case studies and review articles.^1,3,4,6^ Empirical research on HMV focuses primarily on patients' experience of becoming dependent on a ventilator,^7,8^ their experience with health care,^9,10^ meaning of life,^11^ and quality of life.^12^ We also found some studies describing parents' life situation.^13,14^ However, little empirical research exists illustrating the ethical challenges in HMV. The aim of this study was to explore the ethical challenges in HMV, based on a secondary analysis of qualitative empirical data. The study focuses on the ethical challenges related to the most advanced form of HMV, that is, when children and adults depend on tracheostomy, full-time ventilatory support and care to survive. New in our study is how we explored HMV from a broader perspective. The data included healthcare professionals (HCP) in hospitals and community health services and family members of children and adults on HMV.

## Methods

In secondary analysis, data originally collected and analysed in a previous study is re-used to study the previous findings from other perspectives or in order to explore new research questions.^15^ In this study, the collected qualitative data from three previous studies were re-analysed with the use of conventional content analysis. In conventional content analysis, categories (themes) are developed inductively, meaning the researcher is immersed in the data to reveal new insights and to allow new categories to emerge directly from the data.^16^ First, each author analysed the data similarly and independently. During this phase, the transcripts were read and each author isolated major themes describing ethical challenges associated with HMV. Later, the authors met to discuss and agree on the content to be used as the new data source in the remaining part of the analysis process. The text was then grouped in sub-themes, and eventually into a smaller number of themes.

We used 233 pages from 21 focus group sessions and individual in-depth interviews gathered by the research team from three qualitative studies on HMV care between 2008 and 2010 (Table 1). In these studies, we interviewed respondents with different roles in HMV care in Norway: HCP in publicly funded hospitals,^17^ family members of HMV patients (manuscript accepted), and personnel of the publicly funded community health services (manuscript accepted). In the last two mentioned studies, we focused on the respondents’ experience with tracheostomized children and adults with neuromuscular diseases who depended full time on a ventilator and extensive care. In this material, no HMV patients were interviewed.

**Table 1.** Characteristics of the data

Study	Aim of study	Data collection	Data analysis	Respondents	Nr of respondents
1	To find explanations for the huge regional differences in HMV-treatment prevalence.	6 focus groups	Grounded Theory	RNs/Nurses, pulmonologists, anesthesiologists, neurologists, pediatricians, ENT doctor, physiotherapists, medical device technicians.	34
2	To explore the experiences of families giving advanced care to family members dependent on HMV.	10 in-depth interviews	Grounded Theory	Mothers, fathers, wives, husbands, daughter.	15
3	To explore the challenges experienced by health care professionals in community health care services when giving advanced care for patients dependent on HMV.	5 focus groups	Grounded Theory	RNs/Nurses, nurse managers, social educators environmental therapist, child welfare social worker, child pedagogue,personal assistants, certified nursing assistants, licensed vocational nurses.	34

### Ethical considerations

All three original studies were approved by Norwegian Social Science Data Services and the Regional Committee for Medical Research Ethics. All collected material was treated confidentially and all respondents gave their written consent to participate.

## Findings

In the analysis process, we found that the themes related to the ethical challenges dealt with the decision-making process of deciding which patients were to receive HMV care and consideration to the patient’s and the family’s wishes. Respondents also emphasized challenges related to who should make medical decisions in the home and where treatment should take place. Another concern was if and how HMV care could provide both the patient and the family with dignity and increased quality of life. Many of the respondents reflected on what they would have wanted for themselves or their loved ones if confronted with a similar situation. Other challenges related to the implications HMV care had for the patient, family, HCP, equitable distribution of resources and equal access to HMV.

There are many ways to describe and categorize ethical challenges.^18^ We discovered that the main challenges were closely related to the four basic principles of medical ethics: autonomy, beneficence, non-maleficence and justice.^19^ We found it, therefore, appropriate to use these principles as a framework to organize and discuss the findings, even though, during the interviews, we found that these ethical issues were intertwined and often overlapped.

### Autonomy

#### Who decides?

Appointing a medical decision-maker was a central theme in the data material. Hospital HCPs described how ethical challenges related to HMV care were a central theme and were often discussed between involved specialists. General consensus was usually reached for important decisions, but some respondents appealed for national guidelines that could help them prioritize care. They were concerned with considering the patient’s own wishes and the ethical challenges were reduced if the patient made an autonomous decision. Some physicians experienced family members influencing the initiation of HMV or influential family members compelling the patient to decide on initiating HMV treatment, even against the patient’s will:

The family takes charge. I’ve been in situations when you actually know what the patient wants and it was not respected. The patient doesn’t dare stand up against the family because they are so strong. So they make all the decisions. When you are then having a conversation alone with the patient, and you actually know what that patients stands for . . . I have to say, it makes you think. (RN, Focus group 2, Study 1)

Others believed that patients and families did not have the proper medical skills or background to make important and complicated ethical decisions concerning the choice of further treatment options:

I feel we sometimes give too many options when we discuss with them whether they should have a tracheostomy or not. In a way, I think we hand over the difficult issues to the patient and family when there is no reason to, or they do not have the knowledge to do so. (Neurologist, Focus group 6, Study 1)

Family members who had often developed expert competence in HMV and were often involved in daily care at home, wanted to be involved in the important decision-making. They understood the patient's needs best and many ventilator users were too sick to have a complete overview of all decision-making processes, or they were children. Many felt that their homes were invaded by strangers who took charge and got too involved in the daily decisions. The families often felt overrun by the huge communal bureaucracy making decisions the family felt were not for the patient’s or family’s best:

I wore myself out because the community health care services did not have enough people. But they don’t understand this. They follow laws and paragraphs and they don’t see the people involved in this. I would like to go to the media with this. (Mother, Interview 5, Study 2)

For the community health care services, the uncertainty of who was medically responsible was a challenge creating great frustration and frequent conflicts with family members. In these situations, the personnel described this as being rather like finding oneself ‘between a rock and a hard place’: between the patient, family members, and the hospital. The health staff’s professional autonomy was also challenged and influenced because they felt like guests in the patient’s home:

I often have to swallow my pride. It’s their home, and they decide even if I don’t agree, whether to setting boundaries or whatever it may be. They are the parents and as long as it doesn’t affect the life and health [of the patient], I just let them keep on. It’s better than ending up in a huge conflict and destroying the relationship. (RN, Focus group 5, Study 3)

#### What should be decided?

Hospital HCP considered HMV a controversial therapy situated in an ethical ‘grey zone’. The most difficult and most burdensome decisions dealt with whether patients with severe respiratory failure and a poor health prognosis should be offered life-sustaining therapy, which meant choosing between life and death. Decision making, involving patients suffering from ALS or with regard to children with SMA type 1, was highlighted as the most difficult and most controversial:

People have been critical to the scope of help that is needed and which resources are to be used to get this to work . . . people have been critical to initiating all of this for a terminal disease. Imagine a scenario where you could no longer move your eyes, are incontinent, dement . . . when can they then say that we should withdraw treatment. (Neurologist, Focus group 6, Study 1)

In some of these instances, non-invasive ventilation (NIV) was offered. Offering a tracheostomy, when NIV was no longer sufficient, was also a difficult subject. A tracheostomy could extend a patient’s life, but without the guarantee of a good life and with great consequences associated with the use of resources. Disagreements between hospitals concerning the provision of at-home ventilation support to patients with chronic obstructive pulmonary disease (COPD) also ensued. 

In addition to the decision of HMV provision, the most important issue for the family was whether the patient was able to live at home, which the family felt the patient was entitled to according to Norwegian health legislation:

Living at home can be the deciding factor of whether you feel alive or not . . . this is mine. This is my house. And this is where my family lives, as well. It can be just that that makes you want to live. Those provided HMV should be allowed to live at home. (Wife, Interview 8, Study 2)

The biggest challenges facing the community health care services were associated with caring for at-home, high-acuity patients. Frustration with family members due to decision-making and delegation conflicts created an unhealthy working environment leading to HCP quitting after only short periods of time. Some family members also felt that having so many people in and out of their homes was invasive for both the patient and the family:

It’s a good thing to be home, absolutely, but we should look at other alternatives. I’m convinced that some of the parents would benefit from it. I think it could be relieving for them. They could have some time off and forget about who is in the house. (RN, Focus group 5, Study 3)

### Beneficence and non-maleficence

#### What is in the patient's best interest?

In this section, we have decided to merge the findings that can relate to beneficence and non-maleficence. A central ethical theme in the data was whether living with a ventilator was in the patient’s best interest. Alternatively, would discontinuation of treatment constitute a better alternative for a patient with an end-stage disease ending in little functionality and complete respiratory failure if advanced treatment, with often distressing and painful procedures, is not initiated?

The hospital HCP were especially concerned with balancing treatment measures with the question of whether ALS-patients or seriously ill children could have a life with dignity and good quality. It was especially difficult to treat paediatric patients not having the power to consent and in addition relating to parents strongly desiring their child’s survival. In hindsight, and after many years of HMV care, some family members were unsure whether their active role and influence in deciding to initiate treatment was the right thing for the ventilator-dependent family member, especially if the patient were a child:

Have I been too selfish? Is it selfishness that has led us to where we are today? That I am unable to lose her [daughter]? Is that why she is alive today? At the same time, I do talk a lot with her about it. How she’s doing and if she’s feeling ok. Because I am absolutely convinced that when that day comes when she no longer has a good life, then this will no longer be my wish. (Mother, Interview 2, Study 2)

Most of the neurologists interviewed held different attitudes regarding the ethical obligation to provide such comprehensive treatment, because HMV treatment could raise questions regarding the patients’ dignity and quality of life:

They [the neurologists] believe that it is not ethically sound to sustain life with an illness such as ALS, where the person is only able to lie down without the ability to move with the ventilator. The doctor does not think this is a dignified life, and therefore no measures are initiated. This is right. And I think this is a difficult case. (Neurologist, Focus group 4, Study 1)

#### What is in the family's best interest?

The hospital HCP expressed that beneficence does not just concern the patient; the situation for patient’s family members must also be taken into consideration. They believed that HMV care would dominate the lives of the entire family and this was important to consider when making decisions for further treatment strategies:

Yes, I think this is about ethical dilemmas. The efforts made for the child stand in relation to the expected life quality for the child, but also for the entire family. A family perspective is especially important in paediatric neurological habilitation. How does this affect the entire family . . . the siblings, parents . . . the life quality of the child, pain, and distress. How the child is able to develop from these circumstances. Ethical discussions are constantly present. (Paediatrician, Focus group 3, Study 1)

This opinion was a consistent finding when family member experience was analysed. Providing HMV care to high acuity patients had great consequences for the families involved and, in many ways, they had to sacrifice their own lives so that their HMV-dependent loved ones could live a good life. Their biggest challenge involved fighting the system, meaning a community care system unable to provide the high standard of care the families felt was necessary. The families’ presence and expertise was therefore indispensable:

If it wasn’t for me, they would have been overrun. The home health care services make all the decisions. I have to think about my kids. They are helpless. It's all over if the tube falls out of their throats. When the personnel have to work overtime, they fall asleep, and this could prove fatal. (Mother, Interview 5, Study 2)

Comprehensive knowledge and an understanding of the effects that this unique treatment had on the patients, the families, and the community health care services were common attributes shared by all of the respondents. Based on their experience with HMV, the majority of the respondents expressed that they would not choose HMV treatment if their loved ones, or themselves, became ill:

I would not want a hole in my throat and a ventilator. I know how it interferes with family, and the family would be sad if I died before I had to, but just be done with it. It would destroy the family and it would destroy all of us. And it would be a constant grief, instead of dealing with the grief then and there. You’re sad, but to a greater degree done with it and able to move on. (Pulmonologist, Focus group 6, Study 1)

### Justice

#### Resource consequences for other patient groups

Community HCP often brought up the issue of justice when providing highly comprehensive and advanced care to just a few individuals dependent on this type of care. The capabilities of a tight-budgeted community to provide for one or just a few high acuity patients without significant adverse effects on other patient groups became a practical and an ethical question:

In our community these three patients represent 30% of home health care resources and we have almost 800 patients. Having enough personnel is the biggest challenge. It’s a matter of prioritizing in relation to who gets help. And it’s prioritizing we have to do in the community. (RN/ Communal director, Focus group 3, Study 3)

Using financial arguments in the decision-making process, while taking patient rights and what is best for the patient into account, was considered an ethical challenge, distressing, and undesirable for decision-making physicians:

We are accountable to full disclosure in accordance to both patient rights legislation and medical law and we are liable to inform to the best of our ability. And the patient should be able to make a well-informed decision . . . this concerns the patient. I don’t feel it’s right that I should tell the patient . . . to consider community finances in this kind of thing . . . it’s just wrong. (Pulmonologist, Focus group 4, Study 1)

#### Similar cases are treated differently

Hospital HCP constantly referred to the lack of fairness in HMV-provision, because in a few Norwegian counties there were up to seven times more HMV-patients per 100,000 residents compared to other counties with the lowest number of HMV-patients. In this way, some patients were given the opportunity to live while others died earlier on without treatment. The main explanation for the geographical disparities was the dedicated, engaged, and enthusiastic HCP in regions with relatively high numbers of HMV patients. The respondents primarily described this professional enthusiasm as positive and as something the regions with few HMV patients should aspire to achieve. Others believed that enthusiasm could lead to excessive treatment:

If you have an attitude that as many as possible is a good thing, then I think we’ve lost our way. We have to choose the patients carefully, and it’s the COPD group and the neurological illnesses that are most relevant here. We do not have to treat those that are too weak to where it is doubtful that they can come home. I think that’s very important. (Anaesthesiologist, Focus group 2, Study 1)

## Discussion

As previously mentioned, we decided to present our findings in light of the four principles of medical ethics: autonomy, beneficence, non-maleficence and justice.^19^ These principles are not necessarily theoretically comparable to deontological ethics or utilitarianism, but rather used as a practical-ethical framework, and as such have proved influential in medical ethics. Each principle is defined as a duty:^19^

- autonomy: a duty to respect other people's autonomous choices
- beneficence: a duty to do well for other people
- non-maleficence: a duty to avoid harming other people
- justice: a duty to similar handling of similar cases and to allocate resources fairly.

Previous studies show that HMV raises a number of ethical issues about how difficult it is in clinical practice to balance these ethical principles.^3,4,6^ These issues include patient autonomy versus physician paternalism.^5^ In addition, the patient's autonomy should be weighed against the principles of beneficence and non-maleficence;^20^ that is, all the positive and negative aspects in terms of the patient's life condition and presumable future course of the disease. Ethical considerations concerning beneficence and non-maleficence must also include the ventilator-dependent’s family because many aspects of their lives can also become extremely complicated.^14^ The extraordinarily high costs associated with this type of high technological care for a relatively small number of patients, and the wide variation in HMV provision between countries^21^ and within countries,^17^ also raises issues concerning the principle of fair distribution. If ethical challenges are not handled in a proper way, these challenges may negatively affect the quality of care, and represent significant sources of stress for the nursing staff, the patients and the families.^18^

Our findings show that HMV is associated with a wide range of ethical challenges, or dilemmas, on all levels of treatment phases: in the hospital when HMV-provision is decided, for family members living with the HMV-dependent individual and actively participating in HMV care, for the community HCP, and in regards to prioritizing resources at a societal level. In the following, the discussion is limited to the most difficult ethical challenges experienced by respondents. 

### Ethical reflections on HMV and autonomy

The principle of patient autonomy has gained an increasingly prominent role in medical decisions. Such a patient-centred approach seems to stand in contrast to a paternalistic tradition in HMV with the norm being that ‘the doctor knows best’. This could mean that the doctor does not discuss HMV with the patient because the doctor believes that the treatment prolongs the dying process and results in poor quality of life, even when the benefits of HMV are well documented.^22^

In this study, the hospital HCP were determined to act in accordance with the patient’s wishes, but claimed that the patient and the family were incapable of understanding the range of consequences related to their wishes or choices. The ideal of patient participation in medical decision making can be difficult to achieve because of the competence gap in the physician-patient relationship.^23^In our opinion, this stands out as a major concern in relation to the question of life-sustaining HMV initiation, because it might be impossible for the patient and the family to imagine the impact this would have on everyday life. Hospital HCP experienced family members pressuring them to initiate HMV or influential family members compelling the patient to choose HMV against his or her own will. Some researchers have expressed strong concern for physicians making decisions based on the families’ wishes, instead of what the physician considers best for patients unable to make their own decisions.^18^ This can have resource-related consequences; it can damage physicians’ professional integrity and expose the patient to unnecessary treatment.^24^ Initiating HMV against the patient’s wishes may constitute battery, trespass and negligence. It would also be an even clearer violation of patient autonomy, because adult HMV candidates are normally competent to consent. During these value-burdened discussions, HCP must consider that they do not share the same strong, emotional bonds that family members have with their sick loved ones, and that the patient’s life is absolutely the last thing families want to let go.^25^

In ethical considerations, distinction between adults and children is warranted, particularly in relation to autonomy and paternalism. In children, this often leaves the question of who is best suited to determine the best interests for the child.^26^ According to Norwegian legislation, parents have the right to consent to or refuse care on behalf of the child until he or she is at least 16 years old. The Patients’ Rights Act requires that the child should participate in decision making to the extent that it is natural from the child's age and maturity. This study makes one realize how particularly difficult it is to make decisions concerning paediatric patients. Not only were these decisions difficult for the HCP, but also for the parents. After many years with HMV, the parents in our study were unsure whether their initial decision for HMV treatment for the child actually was the best decision for the child. Other studies have also shown that parents have asked themselves whether they made the right decision, but in reality, they did not have other choices if the alternative was to let their child die.^14^ Similar dilemmas are also found in the fields of paediatric and neonatal medicine.^27,28^ Letting their child die is still a choice that is exercised, as painful and harrowing as this is for parents.

Practices concerning the degree to which the families’ wishes are considered in the final decision making differ from country to country.^29^ Norway, like many other European countries, has had a more paternal tradition compared to North America, especially the USA. In recent years, however, there has been a shift toward a more patient-centred approach in European medicine.^29^ Several factors have influenced this development. Increasingly, patients expect to play a much bigger role in treatment and decision making compared to the past. The information age generation are better informed and often explore diagnostic and treatment options prior to consultation with health care services. The Expert Patient movement, such as in the UK, has also empowered patients to have greater involvement in their treatment by taking a leading role in managing chronic conditions to improve health and quality of life and reduce incapacity.^30^

So far, we have discussed issues usually associated with the principle of autonomy. The data material, however, focused more heavily on other and more pragmatic aspects of autonomy. An issue with family members was that HCP made decisions in the families’ private home. On the other hand, the HCP felt that their professional autonomy was threatened because treatment took place in the patient’s private home and family members often made medical decisions. Other studies also report that the nurse’s professional role is challenged when care takes place in the patient’s home.^31^ A possible solution to these problems is to care for the most high acuity patients in assisted-living facilities or nursing homes. This solution, however, could go against the patient’s right to live at home and against the patient’s and the family’s autonomy. Similar to other studies,^32^ most of the family members in our study emphasized that living at home was one of the most important aspects of life quality for the patient and the family.

### Ethical reflections on HMV and beneficence and non-maleficence

The degree of a person’s autonomy varies throughout life and depends on, for example, age and disease. Even though a person’s level of autonomy varies, the person’s dignity and integrity remain constant. Persons depending on HMV care are among the most vulnerable individuals with chronic disabilities.^2^ At a low degree of autonomy, patients are even more vulnerable and dependent on other people in order to maintain their human dignity.^33^

Although the discussion of autonomy is important in our study, beneficence and non-maleficence are perhaps just as important in discussing this issue. These principles are often linked with what is best for the patient. Similar to other ethical considerations, this is especially challenging when concerning children. For example, is a life attached to a ventilator in the best interests of all children? Arguing that treatment is not in the best intererests of specific children stands in contrast to the view that all children have a right to life.^12^ The findings in this study indicate that beneficence and non-maleficence must also include the patient’s family. Hospital and health care services personnel often focused on the burdens the families incurred and this was also confirmed by the family members themselves. These profound consequences, both psychological and physical, also occur with other types of advanced and life-supportive treatments in the home, such as a left ventricular assist device (LVAD).^34^ In our study, consideration with respect to the families was therefore important when hospital HCP were deciding if a patient was to be offered HMV. Most of the hospital HCP, and some of the family members, placed importance on the family’s perspective, in addition to other personal preferences, such as arguing that they themselves would not want HMV. This contrasts with some studies showing that family members, and also HMV patients, would choose HMV again if a similar situation were to occur.^25,35^ This indicates that some people are willing to accept discomfort and heavy burdens for the opportunity to enjoy life. 

Enthusiastic HCP initiate HMV more often than HCP lacking enthusiasm.^17^ From the perspective of beneficence and non-maleficence, the question can be raised of whether it is right that enthusiasts alone should make decisions leading to considerable consequences for healthy family members. It may be easy to be enthusiastic on behalf of others, when you do not feel the physical burden of everyday life experienced by HMV patients and their families. After the loss of an immediate family member who was not given life-extending HMV treatment, the family members have the opportunity to grieve and move on to live normal lives. This study showed that more experienced HCP have become more skeptical and less enthusiastic about the use of advanced HMV having observed the high price the families had to pay. 

### Ethical reflections on HMV and justice

Fair distribution of health care resources is an important societal consideration.^23^ Ethical guidelines for physicians and nurses emphasize the responsible use of societal resources. In a publicly funded health care system, the resources used in one area can negatively affect other patient groups with great needs for treatment and care. Loyalty conflicts can arise when HCP balance the obligations to an individual against societal considerations^23^ or the consideration of one patient up against the considerations of a group of individuals. Should the physician refuse to provide a patient with possible life-extending treatment that could contribute to good life quality, because it is too expensive for the community? Or, in our study, some of the hospital respondents claimed that it was not their responsibility to consider tight community budgets when prioritizing care. But even though society is willing to pay for resource-demanding treatment, there must exist some limits.^36^ Prioritizing is therefore necessary and increasingly more essential due to the growing gap between the population’s expectations, what medical technology can do, and what the economical framework of a publicly funded health care system allows. In 2008, the Norwegian health care authorities signalled a limitation of use of high resource-demanding HMV to ensure a more fair distribution of health care resources. So far, the degree to which these guidelines have been implemented in clinical practice in Norwegian hospitals is unknown. 

In the presentation of the findings, we have mentioned the large geographical differences of HMV-provision in Norway. In regions with relatively many HMV patients, enthusiastic HCP provide treatment. This means that similar cases are not treated equally. This ethical issue has been examined in a previous article^17^ and is therefore mentioned briefly here. 

## Strengths and limitations in this study

This study is one of very few describing the ethical challenges in HMV based on empirical data. We believe that one of the study’s strengths is that the data material is built on the experience of different respondents deeply involved in HMV, both in the hospital and home, and also in families. To gain a broader picture, we could have also interviewed HMV patients, but we chose not to do this, because other studies have already researched life experiences seen from the patients’ perspective.^7,8,10^

In our opinion, this study mirrors the situation in Norway and other comparable European countries. Ethical challenges in HMV will vary between countries and continents due to the differences in medical and cultural traditions, legal and health policy relations, and financing of care. As an example, patient autonomy and freedom of choice are strong traditions in North America, but, in Japan, physicians make the decisions.^29^

## Conclusion

Due to the projected increase of chronic illness, and because development seems to point to the possibility that more diseases and conditions will be possible to treat, one can anticipate that the number of ethical dilemmas in healthcare will grow. Because of high costs, all options will not always be possible to utilize. It is therefore important to put these problem areas on the health policy agenda and encourage more quantitative and qualitative research. It is imperative that decision-makers and those who develop guidelines for prioritizing listen to patients, families, healthcare personnel and researchers.

HMV treatment involves a range of ethical challenges, or dilemmas distributed at all levels of the course of treatment. This study shows how difficult it is to weigh the considerations of the patient against the considerations of the family, health care services, and society. A definition of an ethical dilemma is the lack of a final solution. HMV can be considered as a paradigm case in an ethical dilemma, due to what is possible to achieve using advanced technology and when large human and economical resources are available. Health staff’s enthusiasm should now to a greater degree be weighed in relation to the great personal burden this treatment can lead to for family and next-of-kin.
